# Medical News and Miscellany

**Published:** 1897-05

**Authors:** 


### Medical News and Miscellany.

**Dr. C. T. Cooper** has removed from Alpine to Ballinger, Texas.

---

**Dr. T. E. Standifer** has removed from Tulia, Texas, to Cheyenne, Oklahoma Territory.

---

**The** College of Physicians and Surgeons, of Chicago, has recently become the Medical School of the University of Illinois.

---

**Reminiscences** of Dr. T. G. Richardson. We are indebted to Prof. Souchon, of New Orleans, the author, for a copy of his paper on the life and services of the lamented Prof. Richardson.

---

**Dr. J. Frank Thornton** has removed from Columbus, Tex., to Plum, Fayette, county, Texas. Dr. Lyle Thornton, his brother, delivered the valedictory at Chattanooga Medical College, graduating with third honor.

---

**The Medical Department** of the University of Fort Worth graduated eighteen of their senior class at the annual commencement, held in that city last month. The school is prospering beyond the expectations of its founders.

---

**The Kansas City** *Medical Record* says that the number of medical students in Missouri medical schools has fallen off sixty per cent. this year; and yet St. Louis has a dozen medical colleges that have just *got* to pay expenses.

---

**A physician** practicing in a thrifty village of 400, supported by good farming country, wishes to retire for a year, and travel. He wishes a good physician to go there and take charge of the practice a year, promising to vacate on return of the owner.

The advertiser will stay two months with the new man and induct him into the practice; practice worth from $1000 to $1500 a year. Address of the party can be had by inquiry of the JOURNAL, Austin, Texas. No cost attached.

---

**The N. W. University** Medical School, Chicago, has raised its standard, and the requirements for matriculation and for graduation are now fully equivalent to the requirements of the best colleges of literature, arts or sciences.

---

**Good Location:** A physician, young man preferred, can hear of a location to practice where he can net $100 a month, by writing to Dr. G. L. Jackson, Leakey, Edwards Co., Texas. No regular competition nearer than 23 miles.

---

**Cock's Rational Remedy** for consumption—called, we see, Anti-bacilli Compound,—of which we gave a lengthy account in April issue, is offered to the medical profession through the JOURNAL'S advertising pages this month. See advertisement.

---

**For** non-suppurating buboes of chancrous origin—the injection, into the gland, of a one per cent. solution of benzoate of mercury, first advocated by Welander, is recommended. Welander claimed to have cured without suppuration, fifty-six out of seventy cases.—*N. Y. Medical Record.*

---

GALVESTON, TEXAS, May 6, 1897.

DEAR DOCTOR:—Please state for me in the next issue of the JOURNAL that I lost memorandum of names of two members who paid me their dues on the last day of the Paris meeting, and request the parties to write to me at once, that they may receive proper credit. Truly yours,

H. A. WEST.

---

**Snowed Under.**—Since our editorial on the quarantine question was put in type, the subject of transfer of the State quarantine to the United States government came up in the House of Representatives in the Texas legislature, now in session, in the shape of a proposition to cut off the annual appropriation for quarantine. It was defeated by a vote of 85 to 11. The usual amount of appropriation, $33,000 a year, was agreed upon and reported. At the Paris meeting of the State Association, held on the 27th, 28th, 29th and 30th, ult., an account of which will be found elsewhere, the subject was not referred to, only one of the memorial committee, President Loggins, being present.

---

**Ichthyol in Smallpox.**—"A solution of ichthyol, five or ten per cent., has recently been used with much success as a local application in smallpox, in the pustular stage of the eruption. The solution being painted over the pustules two to four times a day was found to hasten the drying up, check extensive suppuration, and prevent pitting."—*British and Colonial Druggist.*

---

**A Treatment of Epistaxis.**—Mr. Jonathan Hutchinson has made a note in his *Archives of Surgery* of a treatment of epistaxis, which he avers has never failed of success in his hands, and he has had many very rebellious cases. It consists in plunging the patient's feet and hands into water as hot as can be borne.—*New York Medical Journal.*

---

**Not Made in Germany:** Made in America. Arsenauro, the powerful, effective tonic and alterative so useful in obstinate cases of skin disease. Apropos of the very decided penchant that certain doctors have for all foreign-made preparations, we beg to remind them that here is a home-made article of merit worthy of their confidence. See advertisement.

---

**Shrady,** the only "Great American" medical editor,—*mirabile dictu!*—stoops to punning.—Who would have "thunk" it? as Josh Billings would say;—especially as punning is said (vide Lombroso and Nordeau) to be an evidence of degeneracy. Speaking of the tests for urine, Shrady, the only G. A. M. E., says that "Fehling's" test has its "failings," and is not to be relied upon for a delicate test. Now, now, doctor, don't; don't take such an advantage of the unsuspecting.

---

**One** of the pleasant features of the recent meeting of the Texas State Medical Association, at Paris, was the exhibits made by some of the pharmaceutical and instrument houses. The display made by A. P. Cary, of Dallas, was a most creditable one. His line of surgical instruments and appliances was complete in every respect, and he added many patrons to his already large list. Of the pharmaceutical houses the Mellier Drug Company, Parke, Davis & Co., and others were represented, all by courteous, affable gentlemen.

---

**Dr. H C. Black,** of Waco, has been appointed Medical Referee for Texas of the Provident Savings Life Assurance Society of New York, vice Dr. D. R. Wallace resigned. The JOURNAL congratulates Dr. Black on his appointment to this important position, and the Provident Savings Life on securing the services of so competent and trustworthy a man for its Referee.

---

**The** increase in attendance of students at the Medical Department of the University of Texas at Galveston, has been most remarkable, the total number of macrictulants this year being 288. Every department is full; the capacity of the building being taxed to the fullest extent. Think of one hundred and fifty young fellows "cutting up," at one time—I mean —dissecting, and a professor and three demonstrators hopping around from one table to another until midnight every night.

---

**Perennial.**—Jefferson Medical College has decided upon a special spring course in the new laboratories of the college hospital, and is making elaborate preparations for summer laboratory courses and post-graduate instruction. Thus, Jefferson Medical College, says the Philadelphia *Record*, "becomes an institution of perennial value to the country in an educational way." Old Jeff is not going to be left in the rear by younger institutions. Bragg is a good dog: Hold fast is a better.

---

**Consolidation of Schools.**—Bellevue Hospital Medical College and the Medical Department of the New York University have consolidated. The *New York Medical Journal* (April 17, 1897), says this is "a noteworthy token of the tendency that has happily set in [?] to maintain a few schools of unquestionable excellence rather than a greater number of teaching institutions having inadequate facilities."

That such "a tendency has set in" will be news to most people. The tendency seems to be towards a multiplication of colleges, poor in every respect, so long as any three or four persons can constitute themselves a "Faculty," and for $5 get a State charter.

Speaking of this and the consolidation of other colleges, the *New York Medical Record* says that it is done in order to get under the wing of some State university, as the Medical Department, and thus get State aid.

The new concern is to be known as the "New York University-Bellevue Hospital Medical College." The faculty of the new college will number one hundred and sixteen, and there will be ample laboratory accommodations for students in the Loomis and Carnegia laboratories.

---

**The Association's Bill.**—Friends of the Association's bill to regulate the practice of medicine, now in the legislature, and which has so long "hung fire," will be pleased to learn, and we are gratified to be able to state, that on the 4th of May, inst., Dr. Yett, Senator from this (Austin) district, called up the bill and it passed to engrossment, practically without opposition, and, we learn, practically unchanged; the vile amendments to which reference was made in our last issue as having been tacked on, being, on final hearing, voted down. We are encouraged to hope for the final passage of a good medical bill now soon.

---

**College Consolidation.**—As pointed out by the *New York Medical Journal*, there is a general tendency of independent medical colleges to get under the wing of the universities,—to become State institutions. The latest exemplication is the union of the old Alabama Medical College with the University of Alabama; the college becomes the medical department of the University of Alabama. This union was effected at Mobile at the late commencement of the medical college. The Alabama Medical College was founded in 1859 by Dr. J. C. Nott, and a few associates. Nott became the idol of the Alabama profession. Of the first faculty, Dr. Geo. A. Ketchum, the venerated dean of the institution, is the only survivor. He officiated at the late union. May he live to graduate many more young doctors.

We notice in the list of graduates on the occasion referred to the names of two Texas students, Dr. C. W. Cloud, of Burleson, Texas, and Dr. J. T. Seale, of Melrose, Texas.

---

**Decline in the Use of Alcohol in America.**—According to the *American Grocer*, from figures furnished by the United States Internal Revenue and Customs Bureau, there has been a marked decrease in the amount of alcohol consumed in the

United States. The amount of spirits used in 1896, compared with 1888, shows a falling off of nearly one gallon per capita, about 30 per cent. The *Journal of the American Medical Association* is disposed to attribute it largely to hard times. Let us give the "advance of civilization" credit for a part of it, at least; let us hope that man, rational in all else, is awakening to the evil of drink, and is abandoning it. A few years ago it was common to see business men "slightly under the influence of liquor." Now, no self-respecting man drinks, or I may say, no respectable man. The time is coming,—coming fast, thank God,—when the smell of whisky on the breath will be a badge of *odium*, disgrace, which, in time, will lead to social and business ostracism.

---

**For La Grippe.**—A tablet which has been found very efficacious in the febrile stage of la grippe, and which, hereabouts at least, has become generally known as the "grippe tablets," is composed as follows:

℞ Ammonia salicylate..............gr. iii.

Phenacetine.........................gr. 1½

Caffein.

Salicine aa..........................gr. ½ to 1

M. Each tablet contains the above, and one or two tablets constitute an adult dose.

---

**Illinois,** having found the mixed board plan to not work well, is about to get passed now a straight out bill which makes no distinctions as to "schools," but requires the same standard of attainments from all who seek to engage in the practice of *medicine*, i. e., rational therapeutics. That is just right—my idea of a board, exactly. One feature of it, and which, by the bye, is a new departure, is a plan this journal suggested long ago; that is, the State demands and must receive a fee for every license granted upon recommendation of the board. In this case, Illinois, the fee is $50, and the license must be renewed every year; but only $1.00 is charged for renewal. It would seem to be in accord with equity and the general fitness of things that the State should derive revenue from the sale of licenses for this privilege as it does from all other licensed privileges. In that case, however, the State should not expect doctors to make returns of births and deaths, or to render any other service, without compensation. Certainly, the State should not expect a physician to give *an opinion*—expert testimony—at a dollar and a half a day.

### Bureau of Exchange.

A good paying practice in railroad town, black land prairie, Navarro county. Good school and churches. Will induct purchaser into practice. Practice given away to purchaser of residence and office. You will be pleased. Reason for selling, want to move to larger town. Write for terms, etc. Address TEXAS MEDICAL JOURNAL, Bureau of Exchange.

